# Feasibility of a German High-Frequency Neonatal and Pediatric Intensive Care Dataset (AIx-Neo-Guard Dataset): Secondary Data Cohort Study

**DOI:** 10.2196/94280

**Published:** 2026-07-31

**Authors:** Lena Sophie Olivier, Camelia Lauterbach Oprea, André Stollenwerk, Thorsten Orlikowsky, Mark Schoberer

**Affiliations:** 1 Division of Neonatology, Department of Pediatric and Adolescent Medicine Uniklinik RWTH Aachen Aachen Germany; 2 Chair of Embedded Software (Computer Science 11) RWTH Aachen University Aachen Germany

**Keywords:** critical care, database, pediatrics, neonatology, pediatric intensive care units, neonatal intensive care units, mechanical ventilation, data warehousing

## Abstract

**Background:**

Available information about existing neonatal and pediatric intensive care datasets is scarce.

**Objective:**

The objective was to evaluate the feasibility of a monocentric high-frequency dataset of neonatal and pediatric intensive care patients in a cohort study.

**Methods:**

This study included patients treated in the neonatal and pediatric tertiary care intensive care unit at Uniklinik RWTH Aachen, Germany. The dataset comprised high-frequency data on bedside monitoring and ventilation, CO_2_ monitoring, laboratory results, important diagnoses and interventions, patient characteristics, and manual annotations (including procedures, eg, intubation and X-rays; and states and illnesses, such as episodes of apnea of prematurity and patient-ventilator asynchronies).

**Results:**

In total, 400 admissions (227 neonatal and 173 pediatric) from 359 patients were included between September 2024 and January 2026. Of these, 28 neonates were extremely preterm, 34 were very preterm, 68 were moderate to late preterm, and 97 were term infants, with an overall median gestational age of 35 (IQR 31-38) weeks and a median birth weight of 1805 (IQR 1446-3251) g. The pediatric population comprised 34 infants aged younger than 1 year, 45 toddlers aged 1 to 5 years, 43 children aged 6 to 12 years, and 51 adolescents aged 13 to 18 years, with a median age of 10.6 (IQR 1.2-13.0) years and a median admission weight of 22 (IQR 11-50) kg. Overall, 77% (400/517) of all eligible patients were enrolled in the study. The dataset comprised 3600 patient days with more than 20,000 manual annotations (approximately 3 TB) and has been used for multiple use cases.

**Conclusions:**

The high temporal resolution allows for detailed characterization of patient states. To our knowledge, there are no published descriptions of comparable European high-frequency neonatal or pediatric intensive care datasets. We share information about our dataset to encourage cross-institutional cooperation. The multicenter pooling of data or federated learning increases possible use cases by enabling new research questions or the development of more robust algorithms.

## Introduction

Approximately 12% of neonates and 13% of hospitalized children require treatment in an intensive care unit (ICU) [1,2]. Although there have been advances in neonatal and pediatric research in the last decades, many common treatment strategies are still not evidence based [3]. Scientific findings from studies in adult populations are rarely validated on pediatric data [4]. Furthermore, they often cannot be transferred to pediatric patients due to substantial differences in pathophysiology [5]. Despite frequently unknown risks, this results in the widespread off-label use of medication, accounting for up to 50% of prescriptions [6]. This gap of knowledge is particularly problematic because childhood is a critical period for disease development, with long-lasting implications on personal health [7,8].

Neonatal and pediatric intensive care research is challenged by a heterogeneous patient population (eg, with a wide weight range) and small sample sizes [9-11]. Ethical considerations, legal frameworks, and parental uncertainty often obstruct study participation [12]. It can be conflicting to find a balance between protecting the most vulnerable patients by avoiding stress associated with clinical trials on the one hand and obtaining scientific evidence for adequate therapies on the other hand [13]. This leads to recruitment challenges with limited funding and persistently low study enrollment of less than 50% of eligible patients [14-16].

Secondary data research refers to the use of preexisting data that were collected for other purposes and are reused for scientific investigations [17]. It is time-, cost-, and resource-efficient and often faces fewer bureaucratic obstacles than experimental studies [18]. Among others, data can be used for complication detection, clinical decision support, quality analysis, and individualized medicine [19]. It can be helpful in gaining a deeper insight into the effects and side effects of off-label treatments [20]. Despite their advantages and the opportunity to address a variety of unanswered research questions, secondary data are still underused [21].

Data storage solutions for high-frequency intensive care data (such as the Philips Data Warehouse Connect) have been commercially available for almost a decade and are increasingly used. It can be assumed that this is also true for pediatric and neonatal intensive care data, although only very few such datasets are openly available. The storage capacity of these systems exceeds that of *conventional* patient data management systems (PDMSs) by several orders of magnitude. It allows for storing not only numerical but also waveform data at sampling frequencies up to 500 Hz. In turn, this enables researchers to reconstruct critical clinical situations at a high temporal resolution. We established a Data Warehouse Connect system and conducted a study of our pediatric and neonatal patient cohort beginning in August 2024. With regard to data composition, pediatric and neonatal intensive care treatment is characterized by above-average lengths of stay and relatively low patient counts [22]. This poses a dilemma for data research: the limited patient numbers demand cross-institutional data pooling for many research questions [23]. At the same time, low patient counts make it particularly difficult to comply with data protection requirements for anonymized data such as k-anonymity [24]. Our institutionally approved data protection concept therefore demands only sharing anonymized data with third parties for defined purposes with a restricted user circle under contractual protection. The execution recommendations of the Technology and Methods Platform for Networked Medical Research e.V. also advise against the disclosure of anonymized data in public-use files unless “based on general life experience or the current state of scientific knowledge, it is practically impossible to link the data to a specific individual” [25]. The primary objective is to publish the dataset. We hope that, as we move closer to concepts such as the European Health Data Space—including the needed underlying legal perspective—we will be in a position to make the data available to the community in the future. Until then, we see collaborative research as a viable option. This approach allows AI algorithms to be trained independently at different locations using locally available data, such that only model parameters are shared and no explicit patient data are exchanged; this approach is known as federated learning [26]. Another option is to perform external validation for the developed algorithms.

We present our project and metadata with two objectives: (1) to enable potential collaboration partners in our field to determine the extent to which our data and research activities align with their own and (2) to demonstrate that meaningful secondary data analysis can be conducted under the challenging conditions of contemporary neonatal research, even in single centers, and to motivate others to engage in this field [27].

## Methods

### Study Design

AIx-Neo-Guard is a collaborative project of the neonatal and pediatric ICU of Uniklinik RWTH Aachen, Germany, and the Chair of Embedded Software of RWTH Aachen, Germany. The project is conducted as an observational cohort study and was prospectively registered at the German Clinical Trials register (DRKS00033228). The study is conducted in accordance with the Declaration of Helsinki [28] and the STROBE (Strengthening the Reporting of Observational Studies in Epidemiology) statement guidelines for reporting observational studies [29]. Data collection started in September 2024 and has no scheduled end date.

### Ethical Considerations

The study was approved by the local ethics committee (Ethik-Kommission an der Medizinischen Fakultät der Rheinisch-Westfälischen Technischen Hochschule Aachen [RWTH Aachen]; approval EK 24-213). The German Health Data Utilization Act grants an exemption from individual parental informed consent, as only data that are regularly used for diagnostic and therapeutic purposes are collected. Openly available information about ongoing studies at an institution needs to be provided to enable the exemption, and parents can actively revoke study participation.

### Setting

Data are collected at the neonatal and pediatric tertiary care ICU of Uniklinik RWTH Aachen, Germany. The ward offers 9 beds, each equipped with the capability to provide respiratory support. Annually, approximately 350 patients aged 0 to 17 years are treated on the ward.

### Participants

All eligible patients treated in the ICU are included in the dataset. Patients are excluded if their parents object to data processing or study participation. Furthermore, technical issues can lead to unintended exclusion.

### Variables

The dataset incorporates monitoring and ventilation data, laboratory results, patient characteristics, selected diagnoses and interventions, and manual annotations. An overview is given in Table 1. Waveform data are stored at 125 Hz, except for electrocardiogram (ECG) data, which are stored at 500 Hz. Numerical data are recorded with a sampling rate of 1 Hz.

**Table 1:** Available parameters with their temporal resolution and source.

Category and examples				Availability or resolution		Source
Vital signs						Bedside monitoring
	Heart rate		1 Hz			
	Respiratory rate		1 Hz			
	ECG^a^		500 Hz			
	Chest impedance		125 Hz			
	Body temperature		1 Hz			
	Arterial waveform and pressure		125 Hz			
	Noninvasive blood pressure		Intermittent			
Ventilation data						Ventilator
	Settings					
		FiO_2_^b^	1 Hz			
		PEEP^c^	1 Hz			
	Waveforms					
		Airway pressure	125 Hz			
		Airway flow	125 Hz			
	Measurements					
		Measured PEEP	1 Hz			
		Tidal volume	1 Hz			
CO_2_ monitoring						Bedside monitoring
	End-tidal		62.5 Hz			
	Transcutaneous		1 Hz			
Laboratory results				Intermittent		PDMS^d^
	Blood gas analyses					
	Blood count					
	Serum chemistry					
	Urine analyses					
Patient characteristics				One time		PDMS
	Sex					
	(Gestational) age					
	Admission weight					
	Umbilical cord pH					
	Maternal age					
Manual annotations				Partially		PDMS and manual data analyses
	Apnea of prematurity					
	Times of medical imaging					
	Nursing					
	Important interventions					
	Intubation					
	Time of death					
	Patient-ventilator asynchrony					
Body positioning				Partially		Paper documentation
	Prone position					
	Kangaroo care					

^a^ECG: electrocardiogram.

^b^FiO_2_: fraction of inspired oxygen.

^c^PEEP: positive end-expiratory pressure.

^d^PDMS: patient data management system.

### Data Sources and Management

The bedside monitoring is conducted using a Philips IntelliVue X2 monitor (all Philips devices provided by Philips GmbH Market DACH) connected to an IntelliVue MP70 monitor. A Sentec Digital Monitor with V-Sign sensor (SENTEC AG) is used for transcutaneous CO_2_ monitoring. Ventilatory support is supplied by a Leoni plus or Elisa ventilator (Löwenstein Medical). The ventilators and CO_2_ monitoring are connected to the bedside monitoring using Philips IntelliBridge EC5 ID modules connected via a local area network cable to IntelliBridge EC10 modules. EC10 modules are connected to the monitor by a module rack M8048A. All monitoring and ventilation data are retrieved after storage from the Philips Patient Information Center iX using Philips Data Warehouse Connect, version C.03.

Parameters derived from the PDMS (CGM Medico [CompuGroup Medical SE and Co KGaA]) are extracted manually and retrospectively added to the dataset. These comprise patient characteristics (eg, admission weight and sex), diagnoses, laboratory results, body positioning, and interventions (imaging, intubations and extubations, operations, and nursing care). Results from manual data analyses are annotated using a project-intern visualization and annotation tool (refer to the IntensiView Tool section). These annotations included patient-ventilator asynchronies, apnea of prematurity, exact time of blood sampling, disconnection of ventilators or monitoring devices, or suctioning.

**Figure 1:**
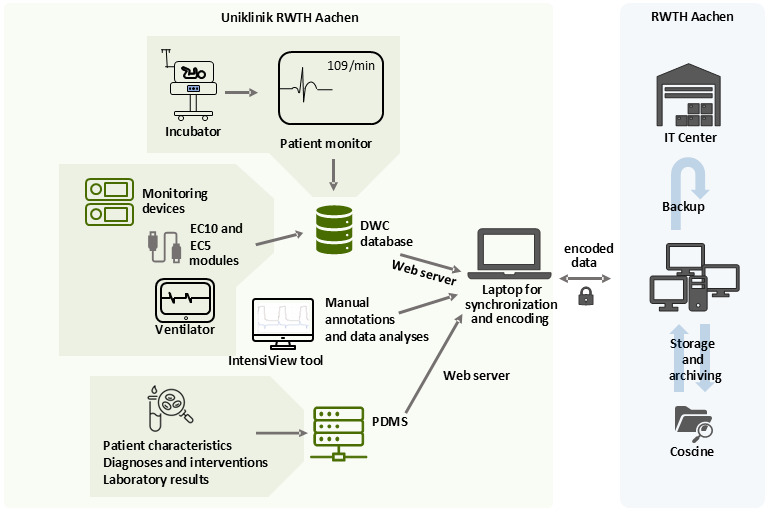
Data flowchart. DWC: Data Warehouse Connect; PDMS: patient data management system; RWTH: Rheinisch-Westfälische Technische Hochschule.

All data, including timelines, are stored in a coded form in a database hosted by the Chair of Embedded Software. The hospital intern patient identifiers are encoded using a randomly generated 32-character universally unique identifier. The encoding table is stored in an access-protected file at Uniklinik RWTH Aachen and only accessible to clinical investigators. A data flowchart is provided in Figure 1.

### IntensiView Tool

**Figure 2:**
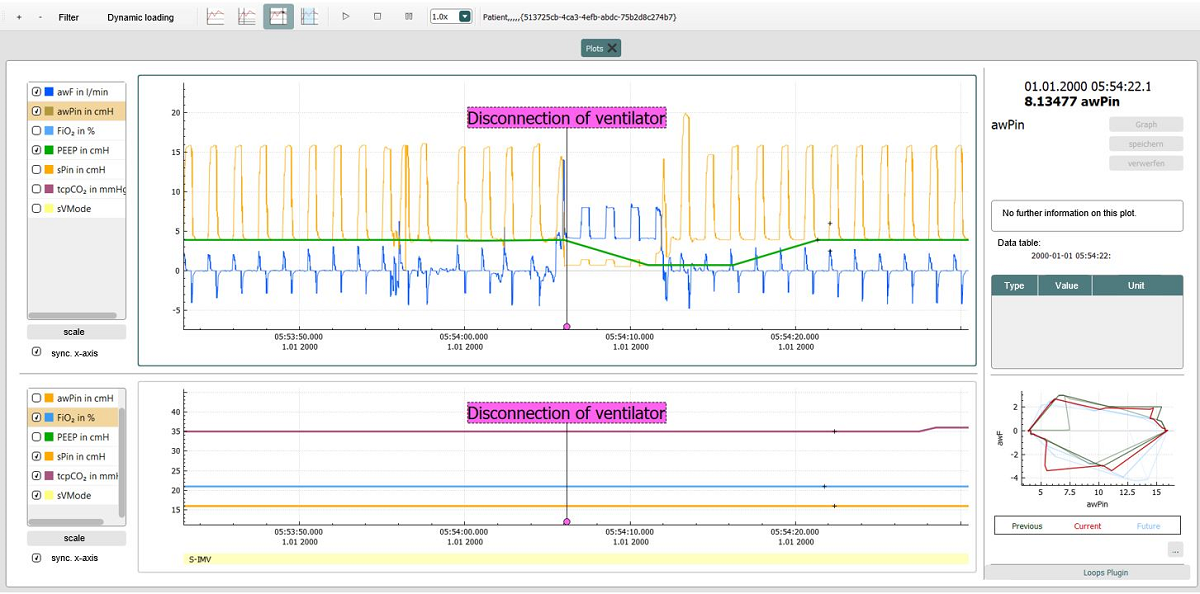
Screenshot of the IntensiView tool for the visualization, annotation, and export of clinical data.

The IntensiView tool (RWTH Aachen University) is a project-specific software developed by the Chair of Embedded Software. The tool is developed in C++ using the Qt Framework to easily deploy across operating systems. A PHP-based backend is used to communicate with the database. IntensiView is used for data synchronization, visualization, annotation, and export. Visualization can be adapted to the user’s requirements by changing colors, adapting the number of plots with freely selectable axes, and switching between column and line diagrams. The annotations can then be added manually or automatized using detection algorithms and are inserted into the database. A screenshot is shown in Figure 2.

An annotation search tool enables the user to reload episodes with specific annotations directly. All data can be exported in CSV format. Additionally, external time-series data and annotations can be easily imported and visualized.

A plug-in interface offers standardized access to the data loaded in the IntensiView tool to integrate new functionality, such as detection algorithms that support current research questions. The tool offers a variety of plug-ins for data analyses, for example, an ECG analysis tool, parameter calculation (eg, mean and median, calculation of airway volume curve from the flow curve, and calculation of airway resistance and compliance from ventilator pressure and flow waveform data), and the creation of ventilator loops.

### Bias

We included all eligible patients to avoid selection bias. All patients were treated according to local standards. The study participation did not alter diagnostics and treatment. Monitoring and measurements were conducted by the same type of measuring devices in all patients to minimize detection bias.

### Study Size

The aim was to include all neonatal and pediatric intensive care patients at Uniklinik RWTH Aachen. Due to limited staff resources, short-stay patients may sometimes not be included because they are discharged before a clinical investigator can link their patient identifiers with the corresponding patient data.

### Quantitative Variables

The following variables were stored in a categorized fashion: sex, age or gestational age, admission weight, admission weight percentile, and type of annotation.

### Statistical Methods

Descriptive statistics, including medians, IQRs, and frequency distributions, were computed to characterize the dataset. No inferential statistical analyses were conducted. Statistical analyses were performed using SPSS (version 29.0.0.0; IBM Corp).

### Data Access and Cleaning Methods

Only the clinical investigators who were directly involved in patient treatment had access to the linking file with the identifiers and were able to access the decoded data at all times. The other project partners had unlimited access to the coded dataset.

After synchronization, the data were reviewed for completeness and consistency. Data cleaning involved identifying missing or incomplete values, ensuring consistency across variables, and resolving any discrepancies. Data anomalies, such as outliers (eg, *z* scores >3), were detected manually or by statistical means. Where appropriate, missing data, duplicate entries, and outliers were removed prior to analyses.

### Linkage

Data were not linked across multiple databases.

## Results

There were 517 ICU admissions during the study period (17 months as of January 27, 2026). Of these, 117 admissions could not be included due to technical issues (primarily Data Warehouse Connect downtime or faulty linkage between intern patient identifiers and Data Warehouse Connect) or missing linkage between patient identifiers and patient data, resulting in a dataset comprising 400 admissions from 359 individual patients.

In total, 227 admissions were attributable to neonatal patients. Of these, 28 were extremely preterm (<28 weeks), 34 very preterm (28+0 to 31+6 weeks), 68 moderate to late preterm (32+0 to 36+6 weeks), and 97 term (≥37 weeks). In total, 173 pediatric admissions were included (34 infants aged <1 year, 45 toddlers aged 1-5 years, 43 children aged 6-12 years, and 51 adolescents aged 13-18 years).

A total of 57.8% (231/400) of admissions were contributed by male study participants. Of the 359 patients, 31 (8.6%) had multiple admissions and 9 (2.5%) had more than 2 admissions. Mortality was 3.6% (14/400) in the study cohort. Median length of stay was 2 (IQR 1-8) days. In total, approximately 3600 patient days were incorporated in the dataset. Patient characteristics are shown in Table 2.

More than 20,000 annotations were added manually to the dataset. The most frequent annotations are active expiration against the ventilator (n=4083, 20.4%), nursing (n=2926, 14.6%), ineffective efforts during expiration (n=1221, 6.1%), apnea of prematurity (n=1271, 6.4%), and time of medical imaging (sonographies: n=185, 0.9% and X-rays: n=153, 0.8%).

So far, the dataset comprises 3 TB of data (implemented using MariaDB as the database management system) and has been used for multiple research questions. A high time resolution was required to address these subjects. An overview is given in Table 3.

**Table 2:** Patient characteristics.

	Neonatal	Pediatric	Total
Number of admissions, n/N (%)	227/400 (56.8)	173/400 (43.3)	400/400 (100)
Number of patients, n/N (%)	225/359 (62.7)	134/359 (37.3)	359/359 (100)
Number of readmissions, n/N (%)	2/227 (0.9)	39/173 (22.5)	41/400 (10.3)
Gestational age (wk), median (IQR)	35 (31-38)	N/A^a^	N/A
Age (y), median (IQR)	N/A	10.6 (1.2-13.0)	N/A
Male sex, n/N (%)	131/227 (57.7)	100/173 (57.8)	231/400 (57.8)
Length of stay (d), median (IQR)	11.75 (1-12.75)	2 (1-7)	2 (1-8)
Mortality, n/N (%)	10/227 (4.4)	3/173 (1.7)	14/400 (3.6)
Birth weight (g), median (IQR)	1805 (1446-3251)	N/A	N/A
Admission weight (kg), median (IQR)	N/A	22 (11-50)	N/A

^a^N/A: not available.

**Table 3:** Published studies conducted with AIx-Neo-Guard data.

Subject	Data type	Patient group	Number of patients	Number of annotations	Publication
IEE^a^	Airway flow curve	Mechanically ventilated neonates	10	2665	[30]
IEE	Airway flow curve	Mechanically ventilated neonates	10	2665	[31]
Breath classification	Airway flow and pressure curve	Mechanically ventilated neonates	18	6304	[32]
Hypoglycemia and hyperglycemia	ECG^b^ Blood glucose	Neonates	12	251	[33]
Lung model	Peak inspiratory pressure PEEP^c^ FiO2^d^ Respiratory rate Blood gas analyses	Neonates	11	460	[34]
tcPCO_2_^e^	tcPCO2 Blood gas analyses	Neonates	12	172	[35]

^a^IEE: ineffective efforts during expiration.

^b^ECG: electrocardiography.

^c^PEEP: positive end-expiratory pressure.

^d^Fi_I_O_2_: inspired oxygen fraction.

^e^tcPCO_2_: transcutaneous CO_2_ monitoring.

## Discussion

### Key Results

In this collaborative project, we successfully established a monocentric high-frequency database of neonatal and pediatric intensive care patients. The dataset incorporates 400 admissions with a total of 3600 treatment days and more than 20,000 manual annotations. We store data from bedside monitoring, ventilation, laboratory results, patient characteristics, and a variety of manual annotations. There is currently no published description of comparable European high-frequency datasets for neonatal and pediatric intensive care patients.

### Interpretation

There are various existing databases incorporating neonatal or pediatric ICU data. Many of these databases contain patient-level data but do not provide high-frequency data [36]. The Pediatrix BabySteps Data Warehouse is a large database of more than 1 million neonatal patients [37]. It contains patient data and characteristics recorded on a daily basis. Many of these data are also included in our database. We collect a variety of additional variables, many of which are captured with high temporal resolution at the second or subsecond sampling levels.

Previously published intensive care databases have paved the way for secondary data analyses. The most commonly used datasets are the Medical Information Mart for Intensive Care-III (MIMIC-III) and MIMIC-IV databases [38,39]. These databases contain medical and demographic data from more than 350,000 ICU patients and have been used in more than 2000 secondary data analyses [40]. However, pediatric patients only constitute a small proportion of the MIMIC-III cohort and were completely excluded from MIMIC-IV. The MIMIC-III Waveform Database [41] extends the clinical data from MIMIC-III by waveform data from approximately 30,000 ICU patients, including 7870 neonates. However, only a subset of these recordings has been matched with the clinical data from MIMIC-III [42]. Comparing the IDs from the matched subset with the original one, we found only 6 neonatal patients with both clinical and waveform data.

Specific pediatric datasets exist: the Pediatric Intensive Care database, for example, incorporates data from more than 12,000 neonatal and pediatric patients [43]. Among others, it provides vital signs, medications, laboratory measurements, and lengths of stay. However, these are stored at low recording frequencies (ie, at 1-minute intervals). The investigation of pathophysiologic conditions often benefits from much higher data resolutions [44].

An example of such a high-frequency dataset is the Preterm Infant Cardio-Respiratory Signals Database [45]. It incorporates 1.6 GB of high-resolution data of ECG and chest impedance from 10 spontaneously breathing premature infants. However, the small sample size and restricted parameter set limit its potential applications.

For pediatric patients, a Canadian group established a database of approximately 1200 patients treated in a pediatric ICU [46]. The researchers collected data at higher frequencies compared to other available databases. The data were recorded every 5 seconds from the bedside monitors and every 30 seconds from the ventilators. This represents a major advancement for research involving high-frequency data. However, this time resolution does not allow a reconstruction of waveform data such as ECG or the airway pressure waveform. In previous analyses, we developed algorithms for detection of ineffective efforts during expiration. They constitute one of the most prevalent forms of patient-ventilator asynchronies [47] and result in prolonged mechanical ventilation and length of stay [48]. The detection algorithms are based on synchronous breath-by-breath waveform analyses of airway flow, which requires a very high time resolution. To the best of our knowledge, there are no descriptions of neonatal or pediatric datasets that can provide high-frequency data with an equivalent parameter set for a comparable sample size in Europe.

An approach for collecting high-frequency data was developed for The Hospital for Sick Children in Toronto, Canada, establishing a new data collection system for waveform storage and retrieval [49]. The database used (AtriumDB) holds waveforms from more than 5300 patients. The paper focuses on the technical development and compression techniques, giving only a short overview of the available data. Furthermore, only waveform data were stored through the described setup, without additional data relevant for intensive care.

Besides the high-resolution monitoring and ventilation data, the AIx-Neo-Guard dataset incorporates important diagnoses, laboratory results, patient characteristics, and manual annotations. This enables a detailed reconstruction of patient states and dynamic physiological changes. In particular, machine learning approaches benefit from such a dataset. The large number of manual annotations can be used to train detection algorithms for highly relevant complications. Furthermore, the unlabeled data can be used for unsupervised or self-supervised learning approaches to detect anomalies, identify clusters, or train foundation models. The integration of high-frequency physiological signals with clinical variables offers an important platform for clinically meaningful and translational research. The dataset opens important opportunities toward outcome-oriented research and enhances pathophysiologic understanding [50]. For example, using detection methods for ineffective efforts based on waveforms together with information on the patient’s state can deepen our understanding of the effects of patient-ventilator asynchrony on the patient’s outcome.

### Limitations

The most important limitation is the single-center study design. First, it limits the available study population. Furthermore, diagnostic and treatment practices are based on local standards, which may introduce institutional and performance bias. We are preparing to collaborate with other centers to increase the available data and reduce the influence of local practices. Joint research across institutions is also desirable to allow the validation of algorithms (eg, for clinical decision support) on external data. This can contribute to enhancing the generalizability of results derived from a particular dataset.

So far, the dataset does not incorporate all relevant information on diagnostics and treatment. Fluid management and medication, for instance, are not yet integrated into the parameter set. Furthermore, the duration, for example, of nursing care is not labeled. This, in turn, is a current limitation for the possible use cases of the dataset. Predictive modeling and causal inference often require this valuable information to provide reliable estimates. The legal framework and ethics approval permit the storage of all variables related to diagnostics and treatment in the ICU. We extend the number of collected parameters continuously and plan to incorporate fluid management and medication data in the future.

Due to the rapid growth of data, there are insufficient staff resources to manually annotate the full dataset. The annotations are continuously extended and improved to enhance the quality of data. The set of annotations may furthermore also be enhanced by automated annotation using machine learning approaches. As previous analyses specifically focused on respiratory events, respiratory annotations are overrepresented compared to nonrespiratory annotations. These annotations will have to be made prior to use for nonrespiratory use cases. The data quality is furthermore hampered by technical issues. Occasionally, the ventilator fails to connect to the central monitoring interface. Resolving this issue would require restarting the ventilator. However, during mechanical ventilation, such an intervention would not be ethically justifiable because it could jeopardize the safety of critically ill patients. Similarly, the patient identifiers must be linked to the patient data on the bedside monitor to include the patient in the dataset. The limited staff resources do not allow a clinical investigator to be present at all times to carry out this step. This problem therefore systematically affects short-stay patients who require only short-term treatment or observation (such as those with transient tachypnea of the newborn, those recovering from operative procedures, or those admitted for intoxications). These episodes result in missing valuable data. This introduces a relevant selection bias toward more severe and longer-stay patients and has an impact on the representativeness of the dataset. Specifically, the overrepresentation of infants with longer or more severe courses will influence descriptive analyses and downstream modeling based on this cohort. As a result, findings derived from this dataset should be interpreted as reflecting the subset of neonatal ICU patients for whom complete data capture was feasible, rather than the entire admitted population.

Finally, the dataset was established to promote secondary data analyses. Secondary data research is always prone to bias due to unmeasured confounding and missing data. As the data were collected for a different purpose, the available data do not always meet the requirements of a specific research question, for example, if parameters of interest are missing. In these cases, other data sources must be sought. We hope to reduce this source of bias by pooling data with other centers in the future, increasing the set of available parameters, and continuing data collection and annotation. While secondary data research can support evidence-based medicine, interventional trials are sometimes still necessary.

### Future Work

We are currently working on machine learning algorithms to automatically detect and correct time stamps for nursing rounds and blood sampling. These are the first steps toward an automated improvement of data quality, as these interventions inevitably lead to data anomalies, such as noisy ECG.

To further extend the dataset, information from pregnancy medical history is currently being integrated into the database for all neonates. In the future, the dataset will be further enriched with microbiological data (eg, results from blood cultures) and more comprehensive information on fluid management and medication administration. This will help improve the interpretability and increase the number of research questions that can be addressed using the dataset.

By sharing these database descriptions, we hope to encourage cross-institutional cooperation and invite all interested researchers to contact us, for example, to conduct external validation of algorithms. We hope that collaborations will promote the multicenter pooling of data and thereby enhance the available data. Larger study cohorts and datasets can improve statistical power and are particularly important for investigating rare diseases, for which adequate sample sizes are often difficult to achieve in single-center studies.

Using federated learning could also leverage the described dataset without the need for direct data sharing. Federated data access enables privacy-preserving development of AI algorithms across institutions and could create a pathway toward more robust foundation models for neonatal and pediatric intensive care.

### Conclusions

We successfully established a monocentric high-frequency neonatal and pediatric intensive care dataset. The available data have been used for various use cases, with further analyses ongoing. Secondary data research can substantially reduce the need for experimental studies and contribute to evidence-based medicine. We hope that ongoing European Union legislation (European Health Data Space) will create a legal framework for the multicenter pooling of data. This would represent a major advance, as it could rapidly increase the available data, counteract bias, and thereby expand research opportunities and improve generalizability.

## Abbreviations

ECG: electrocardiogram
ICU: intensive care unit
MIMIC-III: Medical Information Mart for Intensive Care-III
PDMS: patient data management system
STROBE: Strengthening the Reporting of Observational Studies in Epidemiology

## Data Availability

Data are available from the corresponding author upon reasonable request.
